# ﻿Unrecognized for centuries: distribution and sexual caste descriptions of the West European *Aphaenogaster* species of the *subterranea* group (Hymenoptera, Formicidae)

**DOI:** 10.3897/zookeys.1153.98297

**Published:** 2023-03-16

**Authors:** Enrico Schifani, Antonio Alicata, Lech Borowiec, Fede García, Vincenzo Gentile, Kiko Gómez, Elia Nalini, Fabrizio Rigato, Sämi Schär, Antonio Scupola, Roger Vila, Mattia Menchetti

**Affiliations:** 1 Department of Chemistry, Life Sciences, and Environmental Sustainability, University of Parma, Parma, Italy; 2 Institut de Biologia Evolutiva (CSIC-Univ. Pompeu Fabra), Barcelona, Spain; 3 Department of Biological, Geological and Environmental Sciences, University of Catania, Catania, Italy; 4 Department of Biodiversity and Evolutionary Taxonomy, Myrmecological Laboratory, University of Wroclaw, Wroclaw, Poland; 5 Blesa 45, Barcelona, Spain; 6 C.so Umberto I, 301, Torre Annunziata, Naples, Italy; 7 Falconera 2, Garraf, Barcelona, Spain; 8 Department of Biosciences, University of Milan “La Statale”, Milan, Italy; 9 Museo Civico di Storia Naturale, Milano, Italy; 10 Dietikon, Zürich, Switzerland; 11 Natural History Museum, Verona, Italy

**Keywords:** Ants, *Aphaenogasterichnusa*, biogeography, Mediterranean, Myrmicinae, Stenammini

## Abstract

There are only two *Aphaenogaster* species from the *subterranea* group in the western Mediterranean: *A.ichnusa* Santschi, 1925, from south-western Europe, and *A.subterranea* (Latreille, 1798), also occurring in central and eastern Europe. Historically, the two species have been widely misunderstood: *A.ichnusa* was long considered a Sardinian endemic subspecies of *A.subterranea*, while its continental populations were misidentified as *A.subterranea* s. str. Recently, *A.ichnusa* was elevated to species rank and its worker caste was redescribed with that of *A.subterranea*, allowing for their correct identification. Yet their distribution was documented in detail only for France and Sardinia. Furthermore, no morphological characters were described to distinguish the males and queens of the two species. By investigating private and museum collections, 276 new records of *A.ichnusa* are provided here and 154 of *A.subterranea* from the western Mediterranean. Additionally, qualitative and quantitative morphological characters were combined to identify their males and queens. We present the new southernmost, easternmost, and westernmost distribution limits for *A.ichnusa*. Based on our results, this species is widely distributed in Italy and Catalonia (Spain), also occurring on several Mediterranean islands, avoiding areas with continental climate and high altitudes. Sicily is the only island to host the less thermophilous *A.subterranea*, which otherwise extends westward to Galicia (Spain). Sympatric occurrence is not rare along the contact zone. Additional natural history observations are reported regarding foraging habits, associated myrmecophiles, habitat preferences, and colony structure in the two species.

## ﻿Introduction

The genus *Aphaenogaster* Mayr, 1853 belongs to the myrmicine tribe Stenammini Ashmead, 1905 (Ward et al. 2015) and currently counts 206 valid species and 16 valid subspecies (Bolton 2023). The true genus *Aphaenogaster* is almost exclusively Holarctic, while the tropical species belong to the “*Deromyrma*” clade that awaits formal separation (Branstetter et al. 2022). Most *Aphaenogaster* ant species inhabit the West Palearctic, where six species groups have been identified (Schifani et al. 2022a).

The *subterranea* group currently includes eight species [*A.epirotes* (Emery, 1895), *A.holtzi* (Emery, 1898), *A.ichnusa* Santschi, 1925, *A.kurdica* Ruzsky, 1905, *A.lesbica* Forel, 1913, *A.maculifrons* Kiran & Aktaç, 2008, *A.subcostata* Viehmeyer, 1922, *A.subterranea* (Latreille, 1798)], mostly residing in the eastern Mediterranean and Caucasus region, where several putative undescribed species also exist (Borowiec and Salata 2017; Schifani et al. 2022a). Ten taxa formerly included in this group that are distributed in the Maghreb region and around Sicily, were recently assigned to the distinct *crocea* group according to morphological and genetic evidence (Alicata and Schifani 2019; Schifani et al. 2022a). The only three North African records of *A.subterranea* are based on outdated taxonomy (Forel 1890; Bernard 1967; Cagniant 1970; Henine-Maouche et al. 2022), and most likely represent misidentifications of species of the *crocea* group (Alicata and Schifani 2019; Schifani et al. 2022a). For instance, a worker labeled *A.subterranea* in the Forel’s collection of Geneve Natural History Museum (AntWeb identifier CASENT0907686) is in fact *A.strioloides* (see Schifani et al. 2021a).

Only two species belonging to the *subterranea* group are recognized in West Europe: *A.subterranea* (Latreille, 1798) (terra typica: mainland France) and *A.ichnusa* Santschi, 1925 (terra typica: Sardinia). The first was described using the spelling “*subteranea*” (Latreille 1798), which was later corrected by Latreille himself (Latreille 1802).

*Aphaenogastersubterranea* has a wide distribution, from Iberia to Anatolia, and north to central Europe, while the range of *A.ichnusa* is restricted to West Europe and does not occur in the Balkans (Borowiec and Salata pers. comm., 2022; MM unpublished data). Until recent years *A.ichnusa* was considered as a Sardinian endemic and a subspecies of *A.subterranea*. Their separation as two distinct species was only recently demonstrated based on worker morphology and mitochondrial DNA (Galkowski et al. 2019). The recent recognition implies that reliable data over the distribution of the two species is scarce in the sympatric range, where they were historically confused. Apart from Sardinia, Galkowski et al. (2019) reported the presence of *A.ichnusa* in Corsica and southern mainland France. More recently, only a few scattered records were published from mainland Italy, Sicily, and mainland Spain (Schifani and Alicata 2018; García et al. 2020; Schär et al. 2020; Zara et al. 2021; Bazzato et al. 2022; Scupola et al. 2022). However, a detailed picture of the distribution of *A.ichnusa* and *A.subterranea* in these regions is lacking.

The investigation of specimens from private and museum collections allowed us to gather reliable distribution data of *A.ichnusa* and *A.subterranea* from the west Mediterranean basin, and to fill the current gaps of knowledge. Among them, we investigated several complete nest samples allowing us to morphologically characterize the sexual castes of the two species and provide tools for their identification.

## ﻿Materials and methods

**Figure 1.**
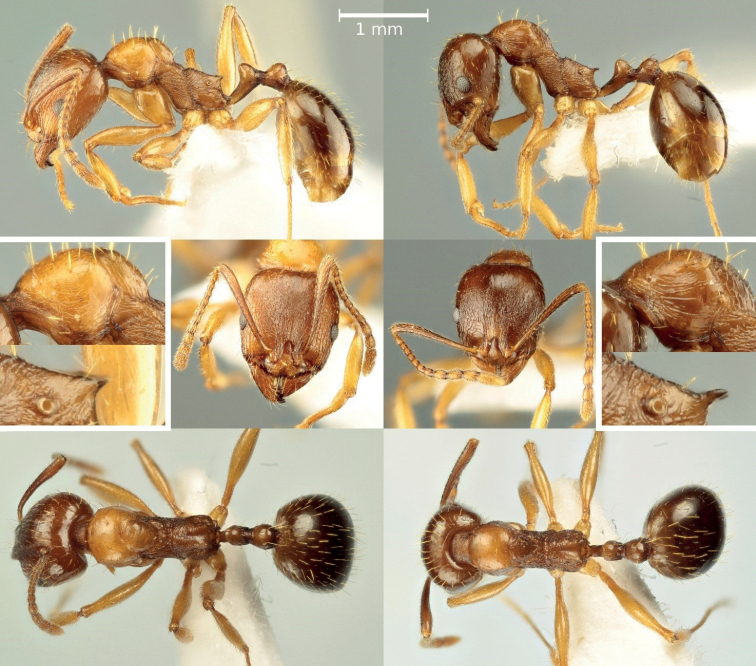
Workers of *Aphaenogasterichnusa* (on the left, sample from Monte Petroso, Sicily) and of *A.subterranea* (on the right, sample from Parma, Italian peninsula). Cropped images highlight the differences in surface sculpturing of the pronotal sides and in the shape of the propodeal spines.

In this study, we focused our investigation on the West European region, where *A.ichnusa* is present and co-occurs with *A.subterranea*. We re-identified worker specimens belonging to the *subterranea* species group found in the authors’ personal collections and the Natural History Museum of Milan. Specimen identification was performed using the morphological characters illustrated by Galkowski et al. (2019): *A.ichnusa* differs from *A.subterranea* in the shorter and thicker propodeal spines, which are triangular, the feebler surface sculpturing of the pronotal sides, and paler pigmentation (Fig. 1).

**Figure 2.**
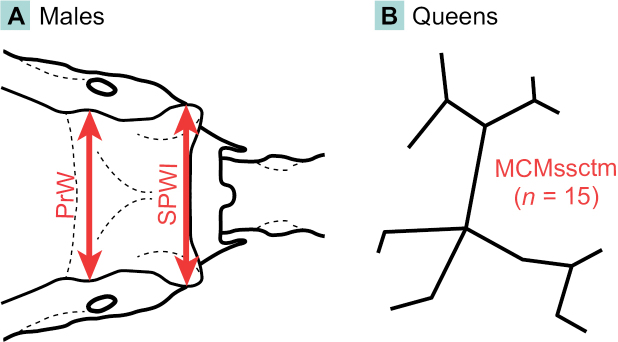
Diagnostic quantitative morphological characters recorded on males (**A**) and queens (**B**) of *Aphaenogasterichnusa* and *A.subterranea*.

Queens and males associated with identified workers were examined to detect further distinctive morphological features. In total, we studied 16 queens from nine colonies and 17 males from seven colonies of *A.ichnusa*, as well as 16 queens from eight colonies and 19 males from nine colonies of *A.subterranea*. Samples were chosen from across the study region and included specimens from their *terrae typicae* (see Suppl. material 1). Due to the unknown, but possibly significant, dispersal capabilities, queens and males unassociated with workers as proof of successful colony establishment were not considered informative for the distribution range of the two species. Morphometric data for the descriptions was produced by taking pictures with a Carl Zeiss Stemi 2000-C stereomicroscope equipped with a CMEX PRO-5 DC.5000p digital camera and measurements taken with ImageFocus 4 software. Three quantitative morphological characters were defined for this study based on those presented by Wagner et al. (2017) and Seifert (2019) (Fig. 2):

**PrW** Males only. In dorsal view, maximum width of the dorsal plate of the propodeum at the level of the propodeal spiracles.

**SPWI** Males only. In dorsal view, maximum width of the dorsal plate of the propodeum at the level of the propodeal spiracles.

**MCMssctm** Queens only. In dorsal view, quantification of stickman-like or reticulate microsculpture units on the mesoscutum: the number of connected lines building units and being separated by line intersections and by flections angled > 30° is counted. Very short lines are also considered full counts. Arithmetic means of three units per specimen are taken.

In addition to means to distinguish males and queens of *A.ichnusa* from those of *A.subterranea*, we provide identification keys that tell them apart from the other *Aphaenogaster* species occurring in the study area (i.e., *A.cardenai*, *A.italica*, *A.ovaticeps*, and species from the *crocea*, *gibbosa*, *pallida*, *splendida*, and *sardoa* groups; see Schifani et al. 2022a).

All characters are given in μm as mean ± sd (min, max). A complete list of the investigated material and of the morphological data is provided in the Suppl. material 1.

## ﻿Results

### ﻿Male diagnosis (Figs 3, 4)

Compared to *A.subterranea*, *A.ichnusa* is characterized by a different propodeal shape with relatively well-developed dentiform spines, as opposed to slight edges. This difference can be appreciated both in lateral view (as the spines protrude backwards from the propodeal profile and are often preceded by a gibbous form in the distalmost part of the propodeal dorsum) and in dorsal view (as the spines are wider than the remaining propodeum). Consequently, the SPWI/PrW ratio was found to be very different between the two species, with no overlap: 1.36 ± 0.14 (1.12, 1.58) in *A.ichnusa*, 0.911 ± 0.06 (0.80, 0.99) in *A.subterranea* (Fig. 4). Furthermore, *A.ichnusa* males are normally substantially darker than those of *A.subterranea*.

**Figure 3.**
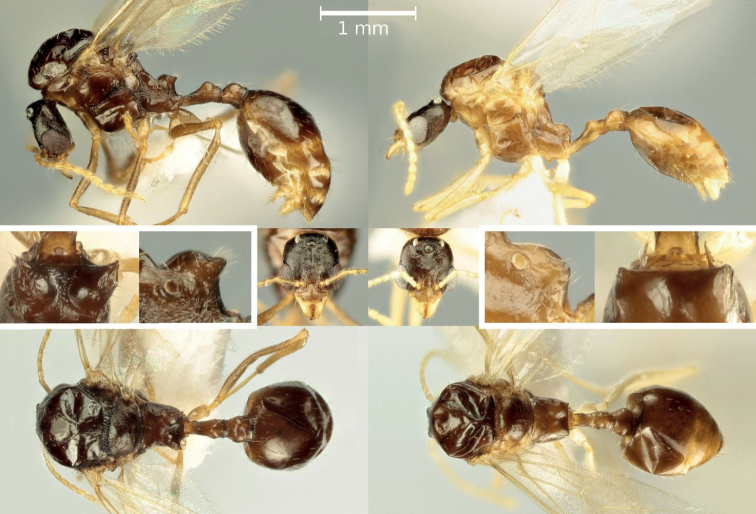
Males of *Aphaenogasterichnusa* (on the left, sample from Monte Petroso, Sicily) and of *A.subterranea* (on the right, sample from Parma, Italian peninsula). Cropped images highlight the differences in the shape of the propodeum.

**Figure 4.**
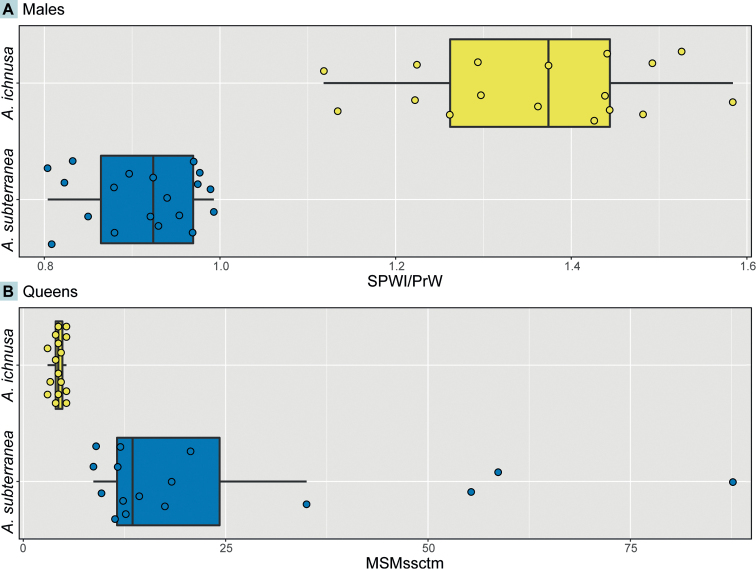
Quantitative morphological differences distinguishing *A.ichnusa* and *A.subterranea* males (**A**) and queens (**B**).

Compared to other sympatric species of the same genus, *A.ichnusa* and *A.subterranea* can be distinguished based on the following combination of characters: shiny integument, mesosoma lacking a pronounced contrast between the anterior gibbosity of the mesosoma and the flat propodeum, the dorsal profile of the propodeum in lateral view relatively short and horizontal, forming approximately a 90° angle with the posterior profile, the rather elongated head shape, the body pigmentation pale to dark brownish.

The following traits are shared by the two species: the head, including mandibles, forms a subtriangular shape; its edges are rounded, and the head capsule is clearly longer than wide. Very large ovoidal compound eyes (~ ½ length of the head capsule) that significantly protrude out of the head capsule, well-distanced from three large ocelli. Mandibles dentate, ~ ½ length of the head capsule, their external side mostly straight for more than ¾. Clypeus medially slightly emarginated. Mesosoma clearly wider than the head, characterized by a well-developed anterior gibbosity. Propodeum much shorter than the remainder of the mesosoma. The horizontal section of the propodeal profile is shorter than its two vertical sections (anterior and posterior), which both form angles of ~ 90° with it. A ventral cuticular process protrudes from the metasternum. Long pedunculate petiole, globose postpetiole, the petiole and postpetiole have a similar heigh, while the postpetiole is wider than the petiole. The legs are long, with hind femurs approximately the same length as the entire mesosoma. The antennae comprise of 11 flagellomeres, the scapi are short, measuring ca. twice the pedicel, and approximately extending slightly beyond the eyes if aligned perpendicularly to the head length axis. Body color pale to dark brown, appendages whitish. Surface sculpturing mostly weak, most of the ant is smooth and shiny, with at most isolated stickman-like units, very feeble rugae or reticulate microsculpture near the sutures, and feeble reticulate microsculpture on the head. Sparse erect and suberect setae mostly occur dorsally over the head, mesosoma and nodes, and both dorsally and ventrally on the first gastral segment and on the distalmost margins of the remaining segments, while shorter suberect or appressed setae cover the appendages.

The following key is meant to facilitate this distinction in the examined region. However, the intraspecific morphological variation of several species is still little known, so we recommend a careful approach in its use.

**Table:** 

1	Notably pronounced contrast between the anterior gibbosity of the mesosoma and a long and relatively flat propodeum, as better observed in lateral view; head less elongated	***A.italica* Bondroit, 1918, *A.sardoa* (Mayr)** (only Sardinia and Sicily), ***A.splendida* (Roger, 1859), *A.gibbosa* (Latreille, 1798) or *crocea* group** (only Sicily and southern Italian peninsula), i.e., ***A.fiorii* Emery, 1915, *A.sicula* Emery, 1908, *A.strioloides* Forel, 1890, or *A.trinacriae* Alicata & Schifani, 2019** (see Emery 1908; 1916; Santschi 1932; Gómez et al. 2018; Alicata and Schifani 2019)
–	Propodeum much shorter and not as flat	**2**
2	Body color blackish; integument either most extensively matt, or mostly smooth and shiny and paired with a wide subrectangular head, or with reticulate microsculpture on the head and visible striae on the mesosoma and petiole	**remaining *A.sardoa* group species** (see Boer 2013), the Iberian endemic ***A.ulibeli* (Gómez et al., 2018), or *A.epirotes*** (in the study area only near the Italian border with Slovenia, see Müller 1923)
–	Body color ferruginous to dark brownish, integument largely smooth and shiny, without strongly developed surface sculpturing	**3**
3	Iberia only, very large size (approximate body length 6–6.8 mm)	***A.cardenai* Espadaler, 1981** (see Tinaut 1986)
–	Combination not as above	**4**
4	Propodeal dorsum in profile view longer and inclined at ~ 45° before reaching the propodeal spiracle, lacking a clearly horizontal component	***A.dulcineae* Emery, 1924, *A.finzii* Müller, 1921**, ***A.ovaticeps* (Emery, 1898), or *A.pallida* (Nylander, 1849)** (Emery 1898; 1916; Müller 1923; Santschi 1932)
–	Propodeal dorsum shorter and more vertical, with a clear horizontal component	***A.ichnusa* or *A.subterranea*** (discrimination possible based on propodeal shape, see characters illustrated in the second paragraph of this section and Figs 3, 4)

### ﻿Queen diagnosis (Figs 4, 5)

Queens of *A.ichnusa* and *A.subterranea* are much more difficult to tell apart based on their general shape as compared to workers and males. However, a subtle but very reliable character was found in the surface microsculpture of the dorsal mesosoma: in *A.ichnusa*, stickman-like reticulations occur sparsely and are modestly developed, while in *A.subterranea* they are visibly more developed and sometimes single stickman-like complexes may connect to each other covering very large areas. MCMssctm ranges do not overlap between the two species, while *A.subterranea* shows a high variation in the upper range: 4.33 ± 0.78 (3.00, 5.33) in *A.ichnusa*, 24.68 ± 22.97 (8.67, 87.67) in *A.subterranea* (Fig. 4).

**Figure 5.**
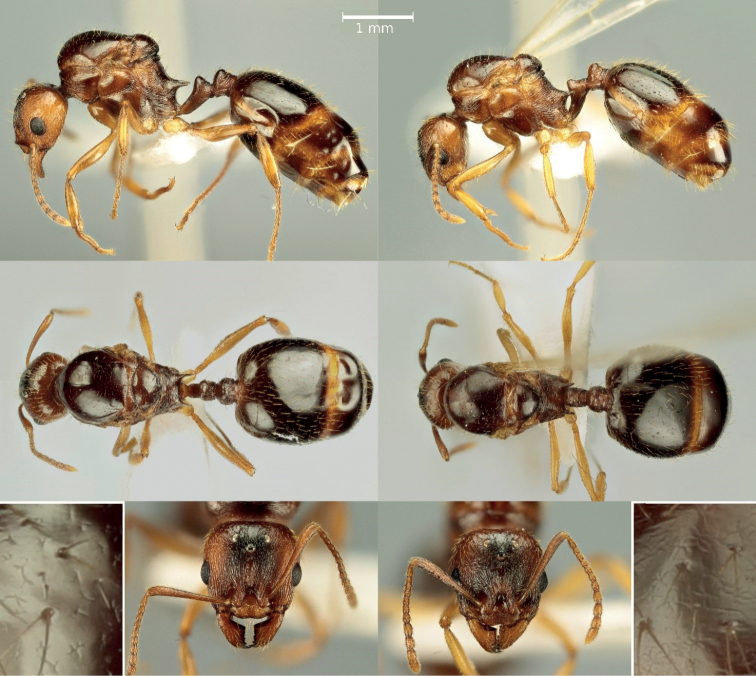
Queens of *Aphaenogasterichnusa* (on the left, sample from Monte Petroso, Sicily) and of *A.subterranea* (on the right, sample from Parma, Italian peninsula). Cropped images highlight the differences in the microsculpture of the mesoscutellum.

Compared to other sympatric species of the same genus, *A.ichnusa* and *A.subterranea* can be distinguished based on the following combination of characters: large mesosoma with wide smooth and shining patches, dark brown to ferruginous pigmentation with blackish areas, body setae relatively sparse, thick propodeal spines and mostly vertical propodeum lateral profile.

The following traits are shared by the two species: The head capsule forms a subrectangular shape, with rounded edges, and is as approximately as long as wide or slightly longer than wide. Large compound eyes (their length ~ ⅓–¼ of the head length) slightly protruding out of the head capsule. The distance between the central among the three ocelli and the level of the compound eyes is ca. the length of the ocellus itself. Mandibles dentate, ~ ½ length of the head capsule, their external side mostly straight for over ¾. Clypeus medially slightly emarginated. Massive mesosoma, slightly wider than the head. Propodeal spines well developed and straight, in profile view the of the propodeum is steep, with the section above the spines slightly inclined and the section below entirely vertical. Long pedunculate petiole, globose postpetiole, the petiole and postpetiole have a similar heigh, while the postpetiole is wider than the petiole. The antennae comprise ten flagellomeres, the scapi are moderately long, surpassing the posterior margin of the head if aligned parallel to the head length axis, while the antennal club consists of four flagellomeres and the flagellomeres 3–8 are only slightly longer than wide. Body color normally not uniform, ferruginous to very dark brown, with blackish patches usually occurring on the frons and paler orangish areas near the sutures of the mesosoma. Appendages pale orange to ferruginous. Rugose sculpture on most of the head, partly on the sides of the mesosoma including the entire propodeum, and on the nodes. The remainder of the body is smooth and shiny, except for stickman-like microsculpture. Abundant erect or suberect setae cover the head, the dorsum of the mesosoma and nodes, the first gastral segment, and the distalmost margins of the remaining segments, while shorter suberect or appressed setae cover the appendages.

The following key is meant to facilitate this distinction in the examined region. However, the intraspecific morphological variation of several species is still little known, so we recommend a careful approach in its use.

**Table:** 

1	Mesosoma matt and smaller, not hosting properly developed flight muscles, all species suspected to be incapable of normal flight dispersal	***sardoa* group** (see Boer 2013; Schifani et al. 2022a)
–	Mesosoma with wide smooth and shining patches and larger	**2**
2	Propodeum longer, its dorsal profile inclined at ~ 45°, propodeal spines thinner, body color uniformly ferruginous	***A.ovaticeps*** (see Salata et al. 2021)
–	Character combination deviating	**3**
3	Body color blackish, striate sculpture on the posterior part of the mesoscutellum and on the metanotum, only near the Italian border with Slovenia	***A.epirotes* (Müller, 1923)**
–	Color paler, mesoscutellum without striae	**4**
4	Body color uniformly very dark brown to blackish, flagellomeres more elongated, head with more parallel sides giving the impression of a more rectangular shape	***A.italica*, *A.gibbosa*, *A.ulibeli*** (see Santschi 1932; Gómez et al. 2018)
–	Body color paler, head sides not as parallel and more convergent frontad	**5**
5	Very hairy, comparatively dense setae at least on the gaster and postpetiole	***A.pallida* group** (*A.dulcineae*, *A.finzii*, or *A.pallida*, see Schifani et al. 2022a)
–	Setae sparser	**6**
6	Body pigmentation yellowish or uniform ferruginous with at most a darkened area on the frons	**either *crocea* group** (only Sicily and southern Italian peninsula), i.e., ***A.fiorii*, *A.sicula*, and *A.trinacriae*, or *A.splendida*** which is normally also characterized by a black transversal band on the first gastral tergite (Alicata and Schifani 2019; Salata et al. 2021)
–	Body pigmentation overall darker and more irregular, blackish patches on the mesosoma and frons	***A.ichnusa* or *A.subterranea*** (discrimination possible based on microsculpture, see characters illustrated in the second paragraph of this section and Figs 4, 5)

Note: The queens of *A.cardenai* and *A.strioloides* are currently unknown and, therefore, they are not included in this key. The first only occurs in Iberia and, considering that its workers are unique among West Palearctic *Aphaenogaster* and that queens usually retain a number of characters of workers, they are highly unlikely to be confusable with species of the *subterranea* group (Schifani et al. 2022a). In West Europe, *A.strioloides* only occurs on the island of Pantelleria, where neither *A.ichnusa* nor *A.subterranea* exist (Schifani et al. 2021a).

**Figure 6.**
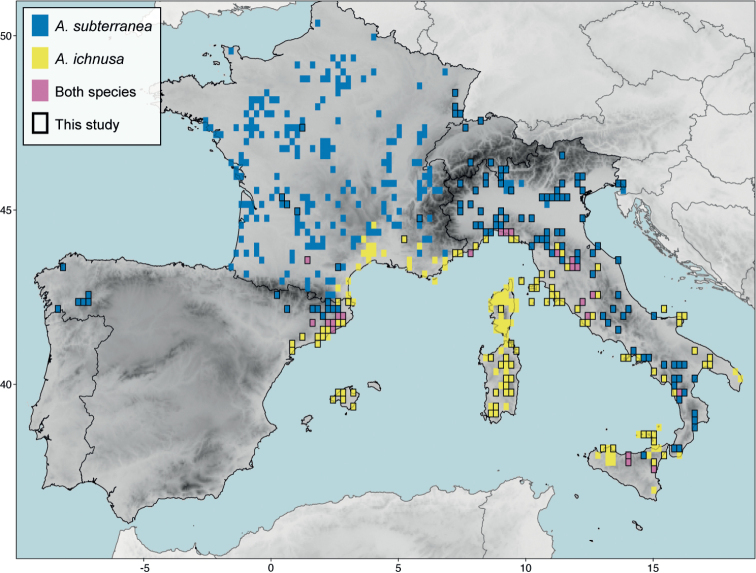
Distribution map showing the locations of the records here newly reported (black border) and retrieved from literature (no border) in a cell grid of 0.2 decimal degrees. Cells are blue for *A.subterranea*, yellow for *A.ichnusa*, and pink if the two species co-occur.

We recovered a total of 274 new records of *A.ichnusa*, and 154 of *A.subterranea* from the study area. We increased the know number of cells of 0.2 decimal degrees (ca. 22 km) occupied by *A.ichnusa* by 145% (103 new compared to 71 from literature) and of those occupied by *A.subterranea* by 64% (130/202). The two species showed a parapatric distribution, with only 4.5% of the cells (22/484) occupied by both species (Fig. 6). The westernmost record of *A.ichnusa* occurs at 0.74 longitude (Catalonia, Spain), the easternmost at 18.46 longitude (Apulia, Italy), the southernmost at 36.94 latitude (Sicily, Italy) and the northernmost at 44.48 latitude (mainland France). For *A.subterranea*, the westernmost record is at -8.46 longitude (Galicia, Spain) and the southernmost at 37.64 latitude (Sicily, Italy).

We report *A.ichnusa* for the first time for several islands and archipelagos: some of the Aeolian Islands (Lipari, Vulcano, Salina, Filicudi, Panarea), the Balearic Islands (Mallorca), the Maddalena Archipelago (Caprera), the Phlegrean Islands (Ischia, Procida), Tavolara island, the Tremiti islands (San Domino), and the Tuscan Archipelago (Capraia, Elba, Giglio, Montecristo).

## ﻿Discussion

The morphological distinction of the *subterranea* group species in the region is relatively easy (Schifani et al. 2022a), and male-based separation of *A.ichnusa* from *A.subterranea* is normally straight-forward as demonstrated by our data. While distinction of queens and workers can be more difficult at first sight, queens are safely separable at adequate magnification and workers are normally easy for a trained eye. Colony samples of *A.ichnusa* with longer spines and colony samples of *A.subterranea* with shorter ones may occur, always requiring a certain attention to other characters such as spine shape, pronotal sculpture and pigmentation. The different ecological requirements of the two species are often helpful but the two may occur sympatrically. While Galkowski et al. (2019) reported a possible introgression event, we did not encounter colony samples with intermediate morphological characters based on subjective evaluation.

While *A.ichnusa* inhabits areas with a Mediterranean climate, *A.subterranea* in the same region occurs only at higher elevations or in specific microclimates. However, *A.subterranea* is frequent at the sea level in continental climatic zones where *A.ichnusa* is absent (e.g., most of mainland France, the Po Plain). We show that *A.ichnusa* is frequent across a vast region of the western Mediterranean (Wang et al. 2023, including Italy’s Tyrrhenian and Ionian coasts, and part of the Adriatic one. Moreover, it is the sole species found on all investigated islands with the only exception of Sicily, where *A.subterranea* is found at higher elevations in the Etna and the Sicilian Apennines alongside other continental species (Schifani et al. 2021b, 2022b).

Notably, both *A.ichnusa* and *A.subterranea* seem to have a more limited distribution in Iberia, where they seem to be restricted to northern Spain. The reliability of few old records from southern Spain appears doubtful in the absence of more recent findings (e.g., Santschi 1919). No historic records exist from Portugal (Arcos et al. 2022), yet our data suggest that targeted investigations may discover *A.subterranea* near the northern border with Spain. The wide distribution of *A.ichnusa* is apparently reflected in the fact that in some old keys, descriptions and drawings attributed to *A.subterranea* were found to depict *A.ichnusa* instead (e.g., Emery 1916 key to the Italian fauna with a drawing of an *A.ichnusa* male). *Aphaenogastersubterranea* may have been temporarily introduced to Madagascar (Csősz et al. 2021).

Colonies of *A.ichnusa* and *A.subterranea* are often found nesting under rocks, and both species may be especially abundant in shady forest habitats with sufficient humidity and leaf litter (Castracani et al. 2010, referring at least part of the records to *A.ichnusa* based on voucher checked; Seifert 2018, assuming Central-European records to represent *A.subterranea*; Zara et al. 2021; Bazzato et al. 2022). Albeit rarely, at least *A.ichnusa* may adapt significantly to urban environments: in summer days, we observed workers foraging in a shady courtyard entirely made of concrete within a historic palace of Florence.

However, despite their ecological success and wide distribution, still relatively little is known ca. the habits of these species besides anecdotal reports. They are probably mostly predators and scavengers, but at least *A.ichnusa* was also observed by the authors to tend root aphids, suggesting that trophobiosis may also play an important role in the diet of both. In addition, we observed isopods (*Platyarthrus* sp.), oribatid mites, silverfish, leaf beetle larvae (Chrysomelidae: Clytrinae), and planthoppers nymphs (Cixiidae) in *A.ichnusa* nests, and isopods (*Platyarthridae* in *A.subterranea* nests, in which also Cixiidae were reported by Lörinczi (2012). In shady habitats, we observed minimal daytime foraging by both species. However, on other occasions, workers of *A.ichnusa* mostly started to forage outside the nest at dusk. Perhaps the foraging activity is often conducted within the leaf litter or in endogean microspaces, but none of these species is truly subterranean (Ortuño et al. 2014).

Some of us also observed cooperative food transport in *A.subterranea* at least. Furthermore, *A.subterranea* workers are known to use tools: they drop small debris into liquid food and transport food-soaked tools back into their nest (Lőrinczi et al. 2018; Módra et al. 2022). This behavior is deemed to be a complex strategy developed to compensate for the inability to ingest and carry into the body large quantities of liquid food (Lőrinczi et al. 2018; Módra et al. 2022). Notably, we found multiple times numerous dealate queens (up to five) in some colonies of *A.ichnusa* and *A.subterranea*, suggesting at least occasional polygyny in both species.

An integrative approach is needed to clarify whether hybridization or introgression between the two species have a significant role as observed in other ants (Seifert 2018). Nuptial flights of *A.subterranea* and *A.ichnusa* seemingly overlap in late summer/early autumn. However, the relatively large size of the contact zone compared to the distribution range of *A.ichnusa*, and the consistent morphological differentiation from *A.subterranea*, suggest some adaptation to counter hybridization. At the same time, the data we are providing could facilitate future studies focused on the ecology and niche partitioning of these species.

## Supplementary materials

Supplementary material 1 Distribution data of *Aphaenogasterichnusa* and *A.subterranea* Data type table (excel document) https://binary.pensoft.net/file/825901This dataset is made available under the Open Database License (http://opendatacommons.org/licenses/odbl/1.0/). The Open Database License (ODbL) is a license agreement intended to allow users to freely share, modify, and use this Dataset while maintaining this same freedom for others, provided that the original source and author(s) are credited.Enrico Schifani, Antonio Alicata, Lech Borowiec, Fede García, Vincenzo Gentile, Kiko Gómez, Elia Nalini, Fabrizio Rigato, Sämi Schär, Antonio Scupola, Roger Vila, Mattia Menchetti
